# The effectiveness of quick starting oral contraception containing nomegestrol acetate and 17-β estradiol on ovulation inhibition: A randomized controlled trial

**DOI:** 10.1038/s41598-020-65642-5

**Published:** 2020-05-29

**Authors:** Preeyaporn Jirakittidul, Surasak Angsuwathana, Manee Rattanachaiyanont, Thunyada Thiampong, Chanon Neungton, Benjaphorn Chotrungrote

**Affiliations:** Department of Obstetrics and Gynecology, Faculty of Medicine Siriraj Hospital, Mahidol University, Bangkok, Thailand

**Keywords:** Endocrinology, Medical research

## Abstract

To determine the effectiveness of quick starting combined oral contraception (COC) contain 2.5 mg nomegestrol acetate and 1.5 mg estradiol (NOMAC/E2) comparing with 0.075 mg gestodene and 0.02 mg ethinyl estradiol (GS/EE) on ovarian ovulation inhibition rate, we conducted a non-inferiority randomized controlled trial involving 69 healthy female volunteers aged 18–40 years who had normal menstrual history and were randomized at a 2:1 ratio to take one pack of COC containing either NOMAC/E2 (study group) or GS/EE (control group) starting on menstrual cycle Day7–9. The ovarian activity was assessed by using Hoogland and Skouby grading. Forty-six and 23 participants were randomized to NOMAC/E2 and GS/EE groups, respectively. Baseline characteristics were similar between groups. No significant difference was observed between the study and control groups for ovulation inhibition rate (93.4% vs. 95.6%, risk difference: –2.2%, 95% CI: –13.1, 8.8), ovarian quiescence rate (91.2% vs. 91.2%, P = 1.000), persistent cyst rate (2.2% vs. 4.4%, *P* = 1.000), and ovulation rate (6.6% vs. 4.4%, *P* = 1.000). Quick starting COC during day7–9 of menstrual cycle can inhibit ovulation for more than 90%. The quick starting NOMAC/E2 is non-inferior to GS/EE for preventing ovulation and suppressing follicular growth.

## Introduction

Combined oral contraception (COC) is one of the most popular forms contraception because of its high efficacy and accessibility^1^. Progestin and estrogen are the two hormonal components of COC. The principle mechanism of contraceptive action is an ovulation inhibition. Progestin compound exerts mainly to prevent ovulation by decreasing LH secretion via a negative feedback on the anterior pituitary gland. In concert, estrogen also acts to prevent the ovulation by negatively influencing FSH secretion and sequel follicular development suppression^2^.

The effectiveness of COC varies significantly depending on its hormonal components and the correctness of use by individuals^3,4^. To achieve the highest contraceptive benefit, the user should start taking the first pill not later than the fifth day of the menstrual cycle, the so-called “conventional start (CS)”^5,6^. However, this starting method may not be practical for women who want to achieve a reliable contraception as soon as possible instead of waiting for their next period. The starting of contraceptive use outside the recommended time is called “quick start (QS)”. This approach is an off-labelled use but it is endorsed by several recommendations^5,6^. Although there is considered to be a small increased risk of pregnancy associated with QS when compared with CS, data specific to QS of COC is scarce. Some studies reported a slightly lower ovulation inhibition rate when QS, with inconclusive data regarding the pregnancy rate compared with CS^7^. Based on our best review, there is little evidence about ovulation inhibition effect of QS of COC use and the emerging information is limited to data from COC containing ethinyl estradiol (EE) with various progestin^8–13^.

Over several decades since the first introduction of COC, various modalities have been employed to produce COC with better tolerability and less cardiovascular adverse effects without compromising the contraceptive efficacy^14–19^. These modalities included the application of newer potent progestin, the reduction of EE dosage, and the replacement of EE with a natural estrogen, 17-β estradiol (E2)^20^. One of the successful COC containing E2 is a monophasic pill comprising 2.5 mg nomegestrol acetate and 1.5 mg E2 (NOMAC/E2) . Although the contraceptive effectiveness of NOMAC/E2 on the ovulation inhibition outcome is reassuring with CS, no data are available with QS practice^19^. Therefore, the present study aimed to determine ovarian activity inhibition associated with QS COCs containing NOMAC/E2 compared with GS/EE.

## Materials and methods

This non-inferiority, single-blinded (investigator-blinded), parallel-arm, randomized controlled trial was conducted at the Family Planning and Reproductive Health Unit, Department of Obstetrics and Gynecology, Faculty of Medicine Siriraj Hospital, Mahidol University, Thailand from April to August 2017.

### Study population

Eligible participants were healthy women aged 18–40 years who had a normal menstrual history (cycle length of 24–38 days). We excluded women who were using hormone within 3 months prior to enrolment, were taking medication with a known interaction with COC, had contraindications to hormonal contraception, had a history of allergy to COC, had a pregnancy, or were undergoing breastfeeding. We further excluded those with body mass index (BMI) of at least 30 kg/M^2^, ovarian cyst/tumor, or pre-existing dominant follicle (leading follicle diameter, LFD, larger than10 mm) at Visit–1. The participants who were sexually active and did not undergo tubal sterilization were instructed to practice sexual abstinence or use a condom throughout the study.

### Study procedure

At Visit–0, each eligible participant was provided with a structure menstrual diary for recording vaginal bleeding pattern for two consecutive cycles. Visit–1 was scheduled for cycle Day1–3 when the participants were interviewed for demographic characteristics, menstrual and obstetric history, and medical and surgical history. The participants then underwent transvaginal ultrasonography (TVUS) to evaluate uterus, adnexa and ovarian follicle diameters. The participants who did not have exclusion criteria were enrolled and appointed for following visits. Visit–2 was scheduled for cycle Day7–9 when the participants were randomized to NOMAC/E2 or GS/EE group in 2:1 ratio using a computer generating random numbers which were individually contained in a sealed opaque envelope; the envelope was serially opened by a research nurse who provided the study COC to each participant accordingly. All participants took their first pill at our clinic immediately after undergone TVUS in this visit and were instructed to take one pill every day at approximately the same time during the study period; if they missed a pill, they had to take the missing pill when they realize that they missed and take the next pill at the regular time; if they missed taking pills more than 24 hours apart, they had to take the next pill as regularly scheduled and they were categorized as protocol deviated participants. A structured patient diary was provided to each participant to record pill-intake, vaginal bleeding, and any adverse effects. Participants underwent repeat TVUS to evaluate follicular growth at Visit-2 and then every 2–3 days thereafter until ovulation or until LFD remained not larger than 13 mm for at least three visits after starting COC, and then every week thereafter until completing the COC package. In each visit, the research nurse checked the patient diary and counted the remaining pills and all participants were asked about the occurrence of any self-reported adverse events.

### Ovarian activity assessment

Ovarian activity was assessed by Hoogland and Skouby (H/S) score which using LFD measurement plus serum estradiol and progesterone levels, as described elsewhere^21^. H/S score were defined in briefly as: (1), LFD ≤ 10 mm; (2), LFD > 10 mm but ≤13 mm; (3), LFD > 13 mm and E2 level ≤ 100 pmol/L; (4), LFD > 13 mm, E2 level>100 pmol/L, and progesterone level ≤5 nmol/L; (5), persisting LFD > 13 mm, E2 level > 100 pmol/L, and progesterone level > 5 nmol/L; (6) ruptured LFD > 13 mm, E2 level > 100 pmol/L, and progesterone level > 5 nmol/L. The ovarian activity in this study was sub-categorized into four groups according to H/S score^21^: (i) *no follicular growth throughout the cycle*, i.e. the score remains 1–2 throughout study; (ii) *regression of ovarian follicle or follicular quiescence in previously growing follicle*, i.e. the score rises to 3–4 and falls thereafter; (iii) *ovulation*, i.e. the score rises to 5–6; (iv) *persisting cyst*, i.e. the score of 3–4 remains at the end of the study and the score has never reached 5–6.

TVUS was performed in every visit using a GE Voluson® 730 Expert Diamond (USA) equipped with a 7.5 MHz vaginal probe. All ultrasound examinations were performed by a single experienced gynecologist (TT) who was unaware of the participant’s allocation. The vaginal probe was placed to the leading follicle as close as possible, in order to make follicle borders visualized clearly. Under the real-time 2D mode, two orthogonal diameters at the largest follicle plane were determined by placing digital calipers at the inner follicle border, then the average value was an LFD which was further used to determine H/S score. The least count of the calipers was 0.1 mm.

Serum estradiol and progesterone levels were checked at each visit when an LFD of at least 13 mm was detected. Serum progesterone level greater than 5 nmol/L was evidence of ovulation. Blood samples were collected into a 5 mL clot-activated tube and sent within 30 minutes to the central laboratory of the Department of Clinical Pathology, Faculty of Medicine Siriraj Hospital, the ISO15189 and DMSC certified laboratory. Electrochemiluminescence competition immunoassay (ECLIA, Cobas) were used to measure serum hormonal levels. The intra-assay and inter-assay coefficients of variation (C.V.) were less than 5%. The minimal detectable limit of estradiol was 55 pmol/L, and of progesterone was 0.6 nmol/L.

### Sample size calculation

This study was using non-inferiority design with a priori to have 80% power and 1-side test alpha 5% to determine the non-inferiority of the study COC (2.5 mg NOMAC and 1.5 mg E2; Zoely®) compared with the reference COC (0.075 mg GS and 0.02 mg EE; Meliane ED®) for ovulation inhibition after QS practice. Based on the previous study that reported a 90% ovulation inhibition rate during QS GS/EE on cycle day 7^8^, we estimated that the study group COC would have the same ovulation inhibition rate with a non-inferiority margin of −20% and 2:1 ratio. The needed sample size for the study and the control groups were 42 and 21, respectively.

### Statistical analysis

Statistical analysis was performed using Stata statistical software (version 11) (StataCorp LLC, College Station, TX, USA). Normality of data was checked using Shapiro-Wilk-W test. Continuous variables were presented in mean and standard deviation (SD) or median and range whereas categorical variables were presented in number (%). Data were analyzed using the t-test or Wilcoxon rank-sum test for continuous data, and Fisher’s exact test for categorical data, as appropriate. Univariable logistic regression analysis was performed to evaluate the difference in ovulation inhibition rate between the two groups which was presented in risk different (RD) and 95% confidence interval (CI) . Cumulative incidence of ovulation was analyzed using Kaplan-Meier survival estimation and Log-rank test for equality of survivor functions. A *P*-value of less than 0.05 was considered statistically significant. Both intention-to-treat (ITT) and per protocol (PP) analyses were applied but only the PP analysis was reported if both analyses provided similar results. Participants who have protocol deviation which including women who took the next pill more lately than 24 hours from the regularly scheduled would be excluded from PP analysis.

### Ethics statement

This study was conducted in accordance with the Declaration of Helsinki with ethical approval by the Siriraj Institutional Review Board COA: Si 804/2016 . The protocol was registered to the ClinicalTrials.gov registry (NCT 03077555, date of registration: 13 March 2017) . All participants provided written informed consents before being enrolled.

## Results

**Figure 1:**
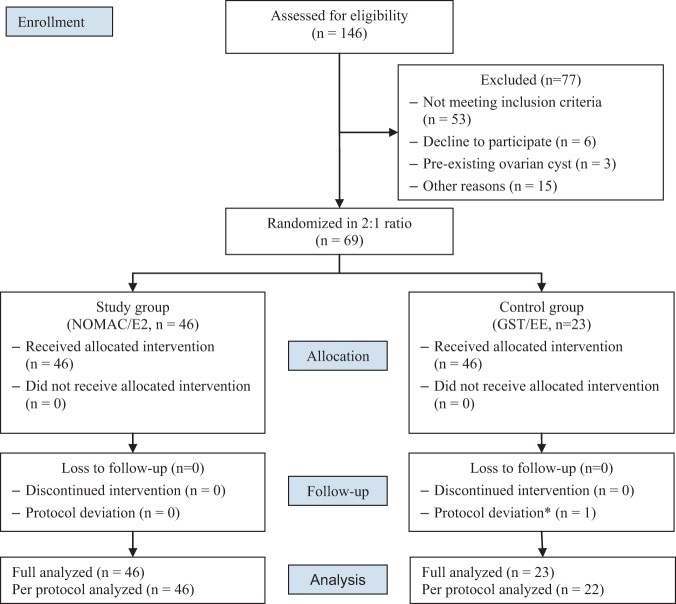
Consort flow chart. *Abbreviations:* *, missed taking pills more than 24 hours apart; GST/EE, 0.075 mg gestodene plus 0.02 mg ethinyl estradiol; NOMAC/E2, 2.5 mg nomegestrol acetate plus 1.5 mg estradiol.

A total of 146 women were screened, 69 women were recruited and randomly assigned to the study group (NOMAC/E2, n = 46) or control group (GS/EE, n = 23). The participants had a mean age of 33.1 ± 5.6 years and mean BMI of 23.4 ± 3.7 kg/M^2^. Of all participants, 27.5% had a BMI greater than 25 kg/M^2^. The average menstrual cycle length was 31.8 ± 3.1 days (range 25–38 days). There was no significant difference between groups for baseline demographic, clinical, or H/S score data (Table 1). Seventy-two percent of participants started taking the first pill on cycle Day-7. The mean LFD on the day of the first pill was 8.8 ± 2.1 mm. Among 69 participants, 52 women had an LFD ≤ 10 mm, 15 women had an LFD > 10 mm but ≤13 mm, and 2 women had an LFD > 13 mm on that day (present as HS 1–4 in Table 1). All of the participants with an LFD not larger than 10 mm at the COC initiation day had successful ovulation inhibition. There were 17 women who had an LFD of larger than 10 mm on the day of the first pill, and four of them ovulated thereafter (3 women with an initial LFD of 14.5 mm, 14.3 mm, and 12 mm in NOMAC/E2 group and 1 woman with an initial LFD of 10.2 mm in GS/EE group) (Fig. S1). One participant in the control group had protocol deviation as she took one pill later than the 24-hour interval, and was excluded from the PP analysis. There was no protocol deviation in the study group (Fig. 1).

**Table 1:** Baseline characteristics of 69 participants.

Characteristics	NOMAC/E2 group (N = 46)n (%) or mean ± SD	GS/EE group (N = 23)n (%) or mean ± SD
Age (years)	33.3 ± 5.3	32.5 ± 6.2
Nulliparous	16 (34.8)	8 (34.8)
BMI (kg/m^2^)	23.0 ± 3.7	23.5 ± 3.7
Menstrual cycle length (days)	31.7 ± 2.9	31.9 ± 3.5
**Menstrual cycle length in each cycle day at starting pill**		
Day-7	31.7 ± 3.2	32.3 ± 3.8
Day-8	31.7 ± 2.4	31 ± 4.2
Day-9	31.5 ± 1.3	30.3 ± 0.5
LFD at starting pill (mm)	8.95 ± 2.2	8.52 ± 2.1
**Cycle day at starting pill**		
Day-7	32 (69.6)	18 (78.3)
Day-8	8 (17.4)	2 (8.7)
Day-9	6 (13.0)	3 (13.0)
**H/S score at starting pill**		
1	35 (76.1)	17 (73.9)
2	9* (19.6)	6^#^ (26.1)
3	0 (0.0)	0 (0.0)
4	2^$^ (4.3)	0 (0.0)

Abbreviations: NOMAC/E2, 2.5 mg nomegestrol acetate plus 1.5 mg estradiol; GST/EE, 0.075 mg gestodene plus 0.02 mg ethinyl estradiol; SD, standard deviation; BMI, body mass index; LFD, leading follicular diameters; H/S, Hoogland and Skouby grading; *, 1 case ovulated (11.1% of women with initial LFD > 10 mm but ≤13 mm at starting pill); ^#^, 1 case ovulated (16.7% of women with initial LFD > 10 mm but ≤13 mm at starting pill); ^$^, 2 case ovulated (100% of women with initial LFD > 13 mm at starting pill).

**Figure 2:**
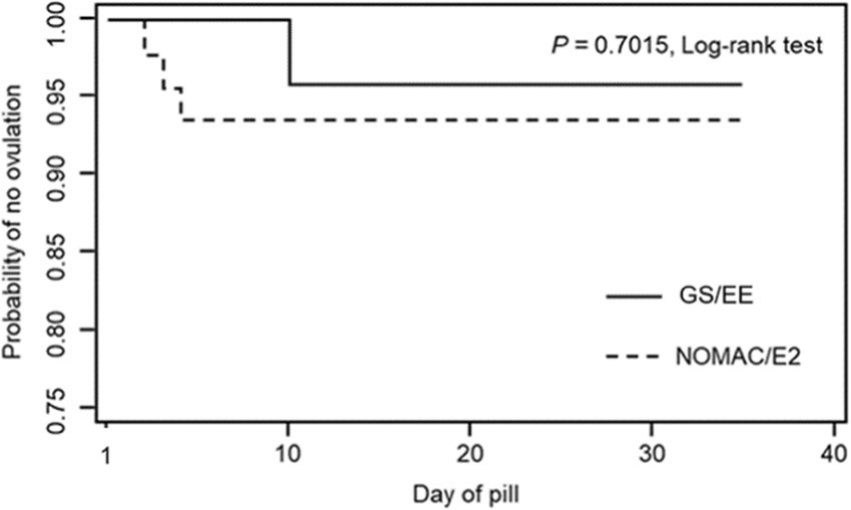
Kaplan-Meier survival curve of per protocol population illustrates cumulative incidence of ovulation inhibition of quick starting combined oral contraception. *Abbreviations:* Day0, day of starting COC; Day 28, day of finishing COC package; GST/EE, 0.075 mg gestodene plus 0.02 mg ethinyl estradiol; NOMAC/E2, 2.5 mg nomegestrol acetate plus 1.5 mg estradiol.

Overall ovulation inhibition was 94.2% (95% CI: 88.5, 99.9); the rate of which was comparable between the study group (93.4%) and the control group (95.6%). There was no statistically significant difference between groups in either ITT analysis (RD: −2.2%, 95%CI: −13.1, 8.8) or PP analysis (RD: −2.0%, 95%CI: −13.2, 9.3) as shown in Table 2. Among the 69 study participants, 4 (5.8%) women had ovulation; three in the study and one in the control groups (Table S1). The ovulation occurred on Day-2, Day-3, and Day-4 of COC in the study group, and on Day-10 of the pill in the control group. The cumulative incidence of ovulation inhibition had no statistical difference between the two groups, P = 0.7015, Log-rank test (Fig. 2).

**Table 2:** Ovulation inhibition outcomes determined using Hoogland and Skouby score*.

	N = 46 n %	N = 23 n %	RD (95% CI)	Adjusted^#^ RD (95%CI)
Intention-to-treat analysis	43 (93.4%)	22 (95.6%)	−2.2 (−13.1, 8.8)	0.3(−10.4, 11.1)
Per protocol analysis	43 (93.4%)	21 (95.4%)	−2.0 (−13.2, 9.3)	0.7 (−10.2, 11.8)

Abbreviations: *, the score never reached 5- 6; NOMAC/E2, 2.5 mg nomegestrol acetate plus 1.5 mg estradiol; GST/EE, 0.075 mg gestodene plus 0.02 mg ethinyl estradiol; ^#^, adjusted for menstrual cycle length, cycle day at starting pill and follicular size at starting pill; RD, risk difference; CI, confident interval.

Table 2 demonstrates ovarian follicular dynamics of women during the course of QS COC. The vast majority of women in both groups had quiescent ovary (either no follicular growth throughout the cycle or regression of ovarian follicle in previously growing one). No significant difference was observed for any follicular development parameters between groups (Table 3).

**Table 3:** Ovarian activity outcomes determined using Hoogland and Skouby score in the intention-to-treat analysis.

	NOMAC/E2 group (N = 46), n (%)	GS/EE group (N = 23), n (%)	*p*-value
No follicular growth throughout the cycle	21 (45.6)	8 (34.7)	0.446
Regression of ovarian follicle	21 (45.6)	13 (56.5)	0.450
Persisting cyst	1 (2.2)	1 (4.4)	1.000
Ovulation	3* (6.6)	1^#^ (4.4)	1.000

A p-value < 0.05 indicates statistical significance

*Abbreviations:* NOMAC/E2, 2.5 mg nomegestrol acetate plus 1.5 mg estradiol; GST/EE, 0.075 mg gestodene plus 0.02 mg ethinyl estradiol; No follicular growth throughout the cycle, the score remains 1–2 throughout study; Regression of ovarian follicle (or Follicular growth prior quiescent), the score rises to 3–4 and falls thereafter; Persisting cyst, the score of 3–4 remains at the end of the study and the score has never reached 5–6; Ovulation, the score rises to 5–6; *, initial leading follicular diameters at starting pill were 12, 14.3, and 14.5 millimeters; ^#^, initial leading follicular diameters at starting pill was 10.2 millimeters.

No serious adverse events (AEs) occurred during the study period, and no participants discontinued their assigned COC. There were 16 AEs in the study group and 12 in the control group. The incidence of AEs was comparable between groups. The most common AEs were unscheduled bleeding (17.4% in the study group and 21.7% in the control group, P = 0.748) and acne (6.5% in the study group and 13.0% in the control group, P = 0.393), as shown in Table 4.

**Table 4:** The self-reported adverse outcomes of quick starting combined oral contraception in 69 participants.

	NOMAC/E2 group(N = 46), n (%)	GST/EE group(N = 23), n (%)	*p*-value
Nausea, vomiting	1 (2.2)	0 (0.0)	1.000
Weight gain	1 (2.2)	2 (8.7)	0.256
Acne	3 (6.5)	3 (13.0)	0.393
Breast pain	2 (4.3)	0 (0.0)	0.549
Unscheduled bleeding	8 (17.4)	5 (21.7)	0.748
Dizziness	0	1 (4.4)	0.333

A p-value < 0.05 indicates statistical significance.

*Abbreviations:* NOMAC/E2, 2.5 mg nomegestrol acetate plus 1.5 mg estradiol; GST/EE, 0.075 mg gestodene plus 0.02 mg ethinyl estradiol.

## Discussion

### Main findings

The present randomized control trial found that the COC containing 2.5 mg NOMAC/1.5 mg E2 was non-inferior to that containing 0.075 mg GS/0.02 mg EE for inhibiting ovulation after quick starting in day7–9 of menstrual cycle. We did not find a difference in ovarian follicular development between groups. Furthermore, there was no statistically significant difference in time-to-ovulation event between the two groups. Our study found no serious adverse event.

### The rationale for choosing the comparison groups

The reasons for choosing the study COC (2.5 mg NOMAC/1.5 mg E2; Zoely®) compared with the reference COC (0.075 mg GS/0.02 mg EE; Meliane ED®) for ovulation inhibition after QS practice in this study were; (1) QS is an off-labelled approach of COC use, but it is endorsed by several standard guideline and recommendations. Additionally, there is little evidence about ovulation inhibition effect of QS of COC use and the emerging information is limited to data from COC containing EE with various progestin (no emerging data from COC containing E2 which may have different impact on ovulation inhibition by an inequality effect on FSH secretion and sequel follicular growth suppression). (2) The only available product of COC containing a natural E2 in Thailand during the studying period was the study COC (2.5 mg NOMAC/1.5 mg E2; Zoely®). (3) The objective of this study was to determine ovarian activity inhibition associated with QS COC containing E2 compared with COC containing EE. Unfortunately, there is little evidence about ovulation inhibition effect of QS of COC use, even from COC containing EE. Based on our review, evidences of QS COC during mid -follicular phase or cycle Day-7 on ovulation inhibition effect were limited to COC containing 0.075 mg GS/0.02 mg EE; Meliane ED®^8^, COC containing 0.150 desogestrel/0.03 mg EE; Marvelon®^13^, and COC containing 0.150 levonorgestrel/0.03 mg EE; Microgynon®^12^ . Data from the study of COC containing 0.075 mg GS/0.02 mg EE; Meliane ED® was the only outcome of QS COC on the fixed cycle day that was similar to our study protocol and the setting of the study was also in Thailand as ours.

### Strengths and limitations

Regarding the strengths of this study; first, this study was a randomized controlled trial, which has low potential biases. Second, the method used to assess ovarian activity (H/S score) is a standard measure for studying the effect of hormonal contraception. Third, the control COC in the present study is a common COC being used worldwide, and the study COC is a novel one containing E2 that is considered safer than the commonly used COC^20^. Therefore, the evidence from the present study is generalizable to women in other countries, and it is the first data of the QS approach in a novel COC with potential safer formulation than the predecessor COCs.

There are mentionable limitations. First, not all participants started their assigned COC on the same menstrual cycle day (range: Day-7 to -9) and these starting days might not be an exact mid-follicular phase in each participant, especially in women who had short or long cycle interval. Second, ovulation outcome was not assessed every day. As such, the reported ovulation day might not be accurate. However, the occurrence of ovulation event was stilled accurately detected with serum progesterone level combined with a serial TVS in this study. Third, the estradiol level could not be used to produce an H/S score in the study group because it contained 17-β estradiol. Forth, although pill count and patient-kept diary can be used for monitoring drug regimens adherence in clinical trial, these approaches are infeasible to assess the exact time of medication taking and of the data entry to the diary. Last, even if the non-inferiority margin was initially set at −20%, it was not prospectively quoted on the trial registration and there was some discrepancy of sample size in the trial registration compared to in the manuscript. Although the total sample size needed in this study was 63, the sample size of 80 was quoted on the registration because the calculating sample size was added about 20–30% for the opportunity of data missing during the follow-up period in this study.

### Interpretation

Ovarian follicles undergo extensive dynamic change during each menstrual cycle. During this period, follicular growth and development were dependent on gonadotropins and steroid hormones. Different and various levels of steroid hormones might have a different effect on follicular cell proliferation, angiogenesis within the follicle and apoptosis. High level of FSH is essential for follicular growth in the initial phase of the menstrual cycle. Generally, during each menstrual cycle, only one follicle is selected to continuing growth and ovulation. At the time of selection, a single dominant follicle was chosen from the cohort of numerous ovarian follicles^22^. Divergence of a potential dominant follicle generally occurred once on cycle Day 6–9 in the early- to mid- follicular phase of menstrual cycle when the follicle size reaches a diameter of approximately 10 mm^7^. Preferential growth of the dominant follicle is associated with an elevation of circulating E2 level, which further provides negative feedback on FSH secretion and inhibit other follicular growth. Continued preovulatory growth of the dominant follicle is responsible for the peak of E2 production and provides positive feedback to stimulate LH surge, which is necessary to induce ovulation^22^.

COC, a combination of estrogen and progestin, is the most commonly used hormonal contraception with a principled action on ovulation inhibition. Progestin suppresses the release of gonadotropins from the anterior pituitary and also decreases the pulse of gonadotropin-releasing hormone secretion from the hypothalamus. An estrogen compound in COC also inhibits FSH secretion by negative feedback to the pituitary and thereby inhibiting follicular growth^2,23^. A Differential steroid hormone compound in COC, such as different type of estrogen or progestin, might have differential effects on ovarian folliculogenesis and ovulation outcome. Additionally, the initiation of the first pill at the first day of menstruation could achieve the highest effect on ovulation inhibition. However, there was considerable evidence that an ovulation inhibition effect was still reliable if start taking the first pill not later than the fifth day of the menstrual cycle, the so called CS.

Quick starting (QS) of contraception, a practice of starting contraception outside the standard timing, may be particularly useful in some women who have long menstrual cycle length, need to start contraception immediately, and may have some barriers to access health service at the later time^6^. QS COC is one of the preferred QS contraceptive methods because it can reduce unintended pregnancy better than emergency contraception, has better continuation rate for later COC use, and has no proven teratogenic effect to the foetus if a pregnancy occurs after starting the COC^5,6^. From literature review, we found no study about QS of COC containing E2. Thus, the main reason for us to perform this study was to prove that the efficacy of QS of COC containing E2 was non-inferior a traditional COC containing EE.

In the present study, an overall ovulation rate was 5.8% (6.6% in the study group, and 4.4% in the control group), which was slightly different from other studies^8,9^. Two previous studies started COC at cycle Day-7 (one study used 0.3 mg norgestrel/0.03 mg EE, and the other used 0.075 mg GS/0.02 mg EE). The study by Schwartz *et al*.^9^ that reported no ovulation in the participants had the too small sample size to yield adequate power for detecting the outcomes of interest; more over the evaluation of ovarian activity using 7-daily ultrasonography was insufficient for detecting ovulation. The study by Sitavarin, *et al*.^8^ that found a 10% ovulation rate after starting COC on cycle Day-7 had comparable ovulation rate to ours. However, the slightly difference in ovulation rate might be contributed by the measurement methods used to detect ovulation. In our present study, ovulation was assessed using the H/S score, a standard measure for ovarian function evaluation in the hormonal contraceptive study, the measure of which employs ovarian follicle ultrasonogram and serum sex hormones to determine ovarian activity.

Corresponding to human folliculogenesis, the ovulation rate during COC use depends on LFD at the time of COC initiation. Ovulation is not suppressed effectively when COC is initiated at the late stage of follicular development when the LFD usually larger than 10 mm^7^. In the present study, no ovulation occurred in the women with LFD not larger than 10 mm at the time of COC initiation, these findings of which were consistent with that of the previous study^13^, and ovulation event was more prevalent in the women who had more larger follicular size at starting the pill. There were 13.3% (2/15) of women who initiated COC use at an LFD > 10 mm but ≤13 mm and 100% (2/2) of women with LFD larger than 13 mm ovulated thereafter in this study. These findings supported the notion that the percentage of cycles where ovulation could be avoided during COC use was inversely correlated with the diameter of a leading follicle at the time of COC initiation. However, the majority of women (13/17; 76.5%) in our study who had an LFD larger than 10 mm at the COC initiation day had follicular regression or anovulation, that was consistent with that of the previous studies which were 64.9%^12^ and 71.4%^13^. These findings supported the use of QS COC for the prevention of unplanned pregnancy by early achieving a reliable contraception as soon as possible and enhancing short-term ongoing use of contraception, the benefit of which was endorsed by several recommendations^5,6^.

Among four women who had ovulation in the present study, three cases had it within the first four days and one case had it on Day-10 of the QS COC. This evidence suggests that women who wish to use QS COC be advised to apply additional non-hormonal contraception, such as condoms or abstinence, during the first 10 days of QS COC at which ovulation can occur. Therefore, the current recommendations that advise women to use a contraceptive back-up method for the first 7 days of QS COC need re-evaluation^5,6^.

## Conclusion

Quick starting COC containing 2.5 mg nomegestrol acetate (NOMAC) and 1.5 mg estradiol (E2) is non-inferior to the one containing 0.075 mg gestodene (GS) and 0.02 mg ethinyl estradiol (EE) for preventing ovulation and suppressing follicular growth in more than 90% of women with a normal menstrual cycle. Ovulation can occur within the first 10 days of the pill, therefore, a back-up contraceptive method is recommended during this period.

## Supplementary information

Supplementary information.

## References

[1] Christin-Maitre S (2013). History of oral contraceptive drugs and their use worldwide. Best. Pract. Res. Clin. Endocrinol. Metab..

[2] D’Arpe S (2016). Ovarian function during hormonal contraception assessed by endocrine and sonographic markers: a systematic review. Reprod. Biomed. Online.

[3] Endrikat J, Gerlinger C, Richard S, Rosenbaum P, Dusterberg B (2011). Ovulation inhibition doses of progestins: a systematic review of the available literature and of marketed preparations worldwide. Contraception.

[4] Morroni C, Findley M, Westhoff C (2017). Does using the “pregnancy checklist” delay safe initiation of contraception?. Contraception.

[5] Curtis KM (2016). U.S. Selected Practice Recommendations for Contraceptive Use, 2016. MMWR Recomm. Rep. 65 No. RR-.

[6] FSRH Quick Starting Contraception. April 2017. Available from, www.fsrh.org/standards-andguidance/current-clinical-guidance/quick-starting-contraception/ [accessed 8 Jun 2017].

[7] Brahmi D, Curtis KM (2013). When can a woman start combined hormonal contraceptives CHCs ? A systematic review. Contraception.

[8] Sitavarin S, Jaisamrarn U, Taneepanichskul S (2003). A randomized trial on the impact of starting day on ovarian follicular activity in very low dose oral contraceptive pills users. J. Med. Assoc. Thai.

[9] Schwartz JL (2002). CM, Pymar HC, Reid L. Predicting risk of ovulation in new start oral contraceptive users. Obstet. Gynecol..

[10] Taylor DR, Anthony FW, Dennis KJ (1986). Suppression of ovarian function by Microgynon 30 in day 1 and day. 5 “starters”. Contraception.

[11] Killick S, Eyong E, Elstein M (1987). Ovarian follicular development in oral contraceptive cycles. Fertil. Steril..

[12] Cameron ST, Berger C, Michie L, Klipping C, Gemzell-Danielsson K (2015). The effects on ovarian activity of ulipristal acetate when ‘quickstarting’ a combined oral contraceptive pill: a prospective, randomized, double-blind parallel-arm, placebo-controlled study. Hum. Reprod..

[13] Baerwald AR, Olatunbosun OA, Pierson RA (2006). Effects of oral contraceptives administered at defined stages of ovarian follicular development. Fertil. Steril..

[14] Burkman R, Bell C, Serfaty D (2011). The evolution of combined oral contraception: improving the risk-to-benefit ratio. Contraception.

[15] Ruan X, Seeger H, Mueck AO (2012). The pharmacology of nomegestrol acetate. Maturitas.

[16] Sitruk-Ware R, Nath A (2013). Characteristics and metabolic effects of estrogen and progestins contained in oral contraceptive pills. Best. Pract. Res. Clin. Endocrinol. Metab..

[17] Stanczyk FZ, Archer DF, Bhavnani BR (2013). Ethinyl estradiol and 17beta-estradiol in combined oral contraceptives: pharmacokinetics, pharmacodynamics and risk assessment. Contraception.

[18] Mueck AO, Sitruk-Ware R (2011). Nomegestrol acetate, a novel progestogen for oral contraception. Steroids.

[19] Akintomide H, Panicker S (2015). Nomegestrol acetate/17-beta estradiol: a review of efficacy, safety, and patient acceptability. Open. Access. J. Contracept..

[20] Del Pup L (2014). Nomegestrol acetate/estradiol hormonal oral contraceptive and breast cancer risk. Anticancer. Drugs.

[21] Hoogland HKSS (1993). Ultrasound evaluation of ovarian activity under oral contraceptives. Contraception.

[22] Baerwald AR, Adams GP, Pierson RA (2012). Ovarian antral folliculogenesis during the human menstrual cycle: a review. Hum. Reprod. update.

[23] Gupta SK, Malik A, Arukha AP (2015). Ovarian and oocyte targets for development of female contraceptives. Expert. Opin. therapeutic targets.

