# Supplementary material for: The effectiveness of quick starting oral contraception containing nomegestrol acetate and 17-β estradiol on ovulation inhibition: A randomized controlled trial
Source: Sci Rep. 2020 May 29;10:8782. doi: 10.1038/s41598-020-65642-5 (PMC7260215; doi:10.1038/s41598-020-65642-5)
Supplement: Supplementary file 1 — Supplementary information. [file 41598_2020_65642_MOESM1_ESM.docx]

**Tittle:**

The effectiveness of quick starting oral contraception containing nomegestrol acetate and 17-β estradiol on ovulation inhibition: A randomized controlled trial

**Authors list:**

Preeyaporn Jirakittidul*, MD; Surasak Angsuwathana, MD; Manee Rattanachaiyanont, MD; Thunyada Thiampong, MD; Chanon Neungton, MD; Benjaphorn Chotrungrote, BNS

*Department of Obstetrics and Gynecology, Faculty of Medicine Siriraj Hospital, Mahidol University, Bangkok, Thailand*

**Corresponding author:**

Preeyaporn Jirakittidul, MD

Department of Obstetrics and Gynecology

Faculty of Medicine Siriraj Hospital, Mahidol University

2 Wanglang Road, Bangkok 10700, Thailand

Tel: (+66) 2-419-4777; Fax: (+66) 2-418-2662

E-mail: [preeyajira@hotmailcom](mailto:manee.rat@mahidol.ac.th)

**Clinical trial registration numbers:** Clinical trials NCT 03077555

**Date of registration:** March 13, 2017

**Supplementary information**

**Table S1.** Characteristics of 4 women who had evidence of ovulation

| Group | Age (years) | Menstrual cycle length (days) | Cycle day at starting pill | Follicular diameter at starting pill (millimeters) |
| --- | --- | --- | --- | --- |
| NOMAC/E2 group | 40 | 28 | Day-7 | 14.5 |
|  | 39 | 30 | Day-9 | 14.3 |
|  | 39 | 26 | Day-7 | 12 |
| GS/EE group | 36 | 28 | Day-8 | 10.2 |

*Abbreviations:* NOMAC/E2, 2.5 mg nomegestrol acetate plus 1.5 mg estradiol; GST/EE, 0.075 mg gestodene plus 0.02 mg ethinyl estradiol

**Figure S1.** The exact size of the follicles when combined oral contraceptive pill was initiated


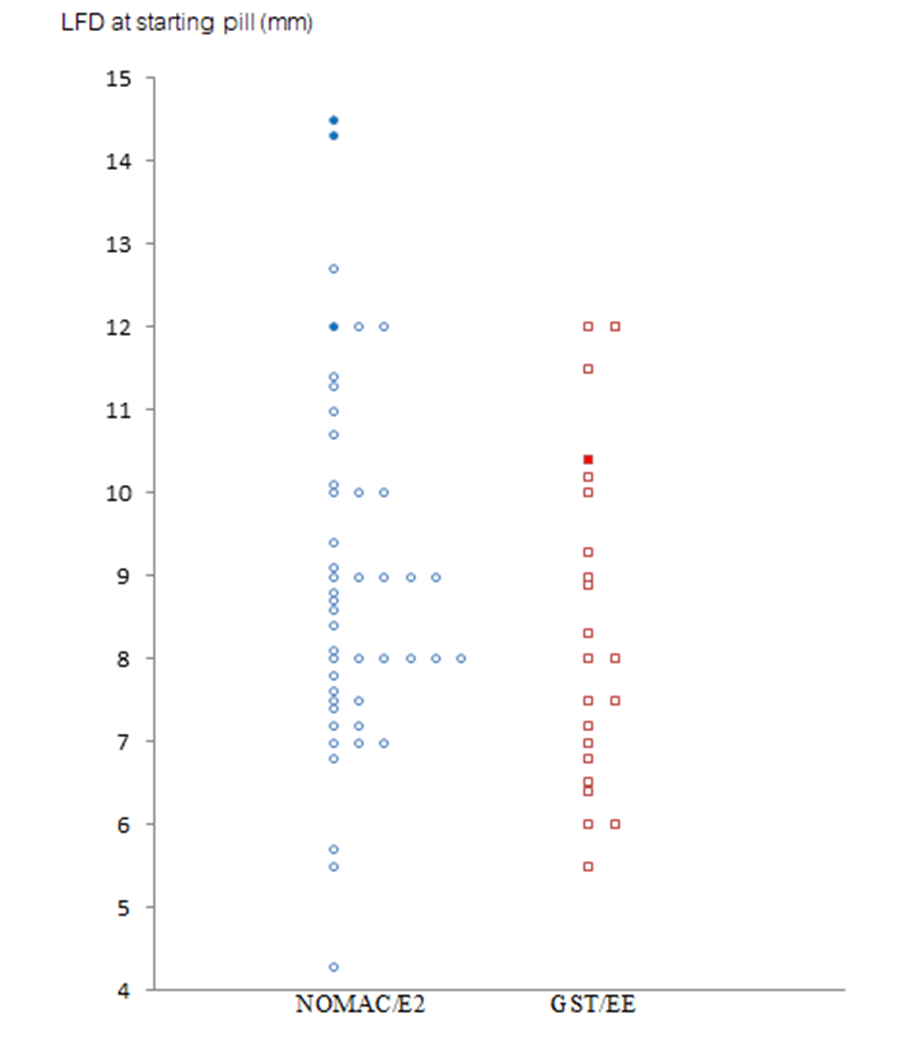


*Abbreviations:* LFD, Leading follicular diameter; NOMAC/E2, 2.5 mg nomegestrol acetate plus 1.5 mg estradiol; GST/EE, 0.075 mg gestodene plus 0.02 mg ethinyl estradiol; solid markers represent the participants who had ovulation occurred
